# In This Issue

**DOI:** 10.1002/cfg.217

**Published:** 2002-12


**Comparative and Functional Genomics**
*Comp Funct Genom* 2002; **3**: 469.
Published online in Wiley InterScience (www.interscience.wiley.com). **DOI:** 10.1002/cfg.217

### In this issue

### Proteome Analysis of the Plasma Membrane of *Mycobacterium tuberculosis*

Sudhir Sinha and colleagues report on their proteomic study of the plasma membrane of the bacteria that causes TB in man. Using 2DGE and MALDI–MS analysis led to the identification of 32 proteins, 17 of which were new to the *M. tuberculosis* proteome database. Where possible, the proteins were then classified as 'membrane-associated', 'membrane-bound' or 'lipoproteins' using a combination of programs.

### Special Section of Selected Reviews from the From Genome to Life International Summer School

Mohamed Zouine *et al.* provide us with a timely reminder of how to use the concepts of homology and classes of homology correctly, illustrating the importance of these concepts with their own work on using intergenomic comparisons of *E. coli*, *C. jejuni*, *H. influenzae* and *H. pylori* to understand the molecular evolution of genes.

Igor Berezovsky and Edward Trifonov have applied the laws of polymer physics to their analysis of protein structure. This has revealed that the basic unit of protein structure is a closed loop of 25–35 amino acid residues with proteins being built of linear sets of these loops.

Konstantin Khodosevich and colleagues discuss endogenous retroviruses (ERVs) and the potential role of human-specific ERVs in the divergence of the human ancestor from those of the apes.

Thomas Schlitt and Alvis Brazma review three recent papers that have built gene regulatory networks based on a microarray dataset of yeast gene deletion mutants. They compare the approaches taken by each group and discuss the conclusions drawn from each study.

Mounia Heddad and Iwona Adamska discuss the evolution of light stress proteins in photosynthetic organisms, analysing the occurrence of Elip family members in various photosynthetic prokaryotic and eukaryotic organisms and discussing their evolutionary relationship with Cab proteins.

Isabelle Mus-Veteau provides an overview of the methods available and the successes so far in heterologous expression and purification of mammalian membrane proteins for structural analysis.

Neocles Leontis and Eric Westhof remind us that there are 12 possible edge-to-edge interactions that can occur between bases to form base pairs in RNA structures. They demonstrate their convention for annotating motifs and show how this nomenclature can be used to summarize RNA tertiary structure in a 2D format.

### Meeting Review: From Genome to Life International Summer School

We present overviews of the main presentations given at the From Genome to Life Summer School. Invited speakers included Eugene Koonin, Stephen Oliver, Steve Brenner, Thierry Rabilloud, Philip Avner and Wolfgang Baumeister.

### Meeting Review: ISMB 2002

Cara Woodwark reports on the keynote presentations at this year's Intelligent Systems for Molecular Biology conference. The keynote speakers included Ford Doolittle, Stephen Altschul, Terry Gaasterland and Overton Prize winner, David Baker.

Copyright © 2002 John Wiley & Sons, Ltd.

